# Supplementary material for: The Na,K-ATPase acts upstream of phosphoinositide PI(4,5)P2 facilitating unconventional secretion of Fibroblast Growth Factor 2
Source: Commun Biol. 2020 Mar 25;3:141. doi: 10.1038/s42003-020-0871-y (PMC7096399; doi:10.1038/s42003-020-0871-y)
Supplement: Supplementary file 1 — Descriptions of Additional Supplementary Files [file 42003_2020_871_MOESM1_ESM.pdf]

Supplementary Movie 1: Atomistic molecular dynamics simulations of the FGF2/α1-subCD3 interface based on the WT1 system.

Comparison of the interaction of FGF2-wt and FGF2-K54/60E with α1-subCD3 using MD simulations. While the FGF2-wt/α1-subCD3 interface (left panel) remains stable during the whole simulation time (only the last 100 ns are shown in the video), FGF2-K54/60E dissociates from the α1-subCD3 domain already during the first 100 ns of simulation. See Materials and Methods for simulation details. The color coding and the representation styles for FGF2 and α1-subCD3 correspond to Figure 6. In addition, the licorice style is used to highlight the residues that are in contact with K54 and K60 from FGF2.

Supplementary Movie 2: Single particle analysis of FGF2 membrane recruitment at the inner plasma membrane leaflet in living cells

Time-lapse TIRF videos (100 ms/frame) of CHO-K1 cell lines expressing the FGF2-GFP fusion proteins introduced in Supplementary Figure 5A and in Fig. 8A (FGF2-K54E, FGF-K60E and FGF2-K54/60E). Background fluorescence was subtracted. Scale bar = 6 μm.

Supplementary Movie 3: Single particle analysis of FGF2 membrane recruitment at the inner plasma membrane leaflet in living cells

Time-lapse TIRF videos (100 ms/frame) of CHO-K1 cell lines expressing the FGF2-GFP fusion proteins introduced in Supplementary Figure 5B and in Fig. 8B (FGF2-K54E, FGF-K60E and FGF2-K54/60E combined with K127Q/R128Q/K133Q). Background fluorescence was subtracted. Scale bar = 6 μm.
