# Supplementary material for: The Na,K-ATPase acts upstream of phosphoinositide PI(4,5)P2 facilitating unconventional secretion of Fibroblast Growth Factor 2
Source: Commun Biol. 2020 Mar 25;3:141. doi: 10.1038/s42003-020-0871-y (PMC7096399; doi:10.1038/s42003-020-0871-y)
Supplement: Supplementary file 6 — Supplementary Information [file 42003_2020_871_MOESM6_ESM.pdf]

Supplementary Figures

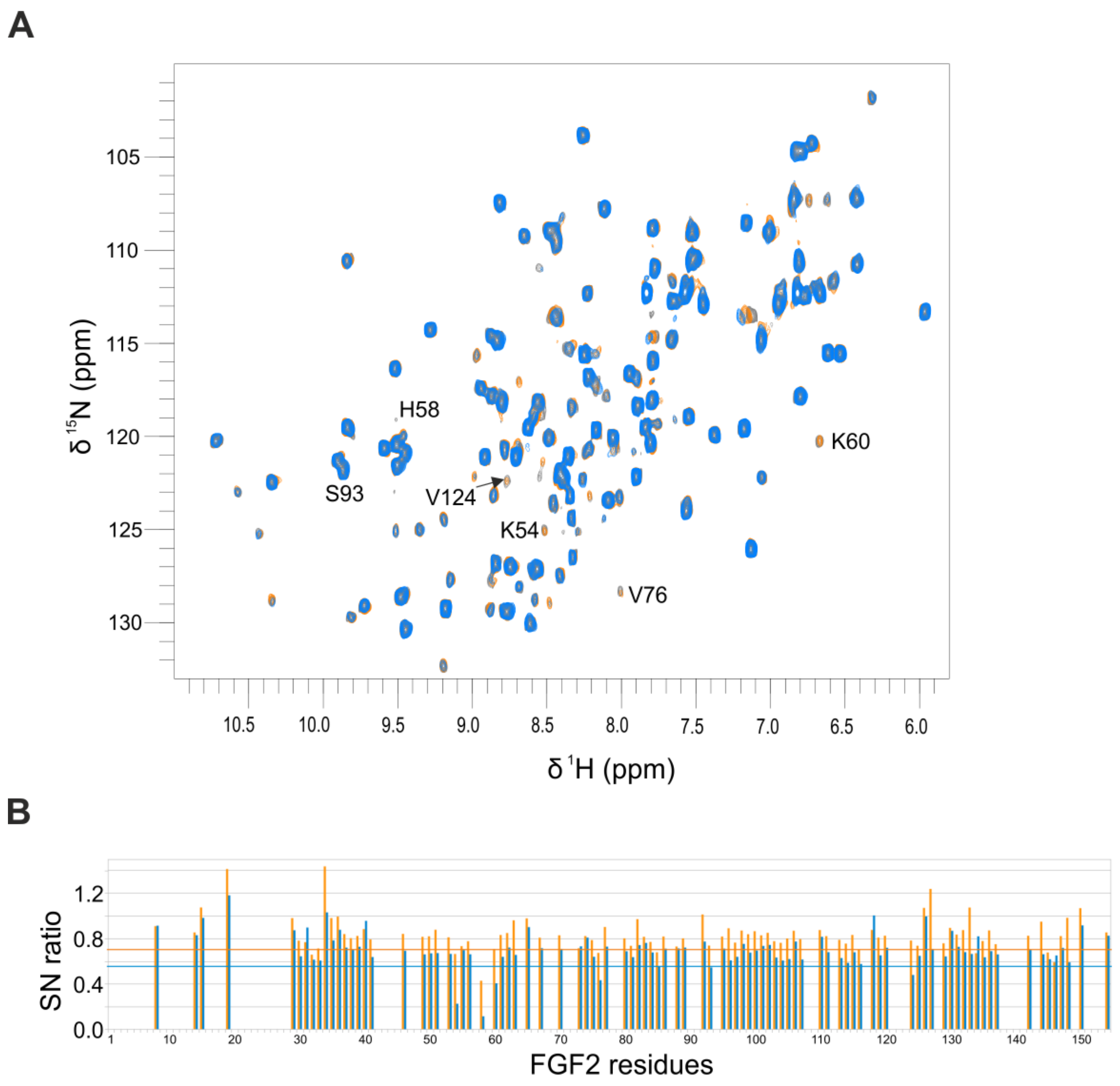

Supplementary Figure 1

A) Shown is the overlay of the ^{1}H-^{15}N-HSQC spectra of 77 µM ^{15}N-labeled FGF2-C77/95S in the absence (grey) or presence of 70 µM (orange) or 138 µM α1-subCD3 (blue). Chemical shift differences of assigned peaks are very small (<0.04ppm), with an average value of 0.007 ppm. Peak assignments are indicated for those peaks showing a reduction in signal intensity upon addition of 138 µM α1-subCD3 (see also B).

B) Plotted are the signal-to-noise ratios (SN ratio) for all assigned peaks with SN(+70 µM α1-subCD3)/SN(-α1-subCD3) in orange and SN(+138 µM α1-subCD3)/SN(-α1-subCD3) in blue. Addition of α1-subCD3 leads to an overall reduction in SN to on average 0.84 (+70 µM) and to 0.70 (+138 µM). A reduction in signal-to-noise beyond the average value minus standard deviation is considered meaningful. This value is 0.70 for the addition of 70 µM α1 (orange line) and 0.55 for the addition of 138 µM α1 (blue line). Overlapping peaks were omitted from the analysis.

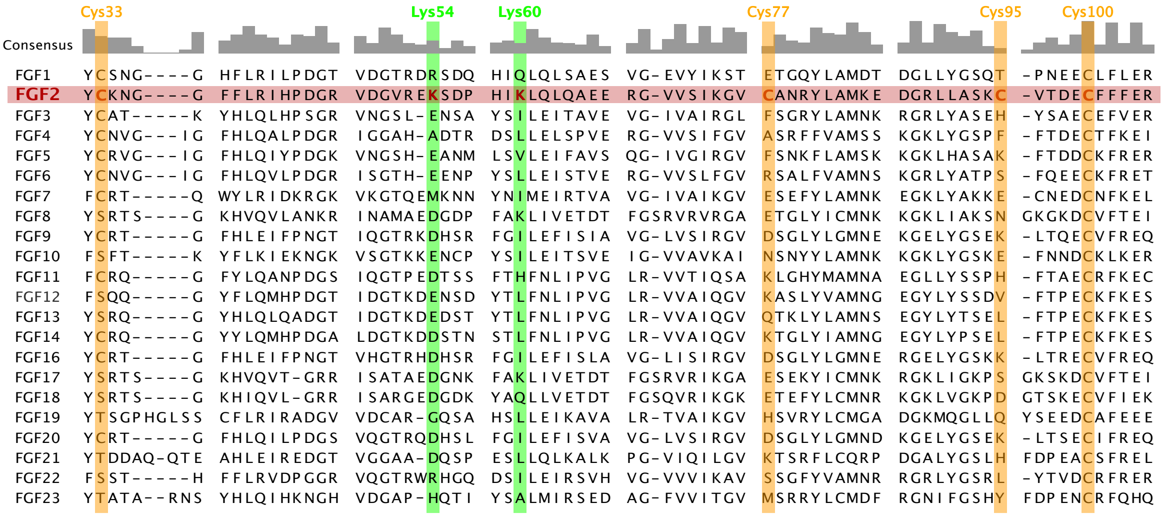

Supplementary Figure 2: Residues critical for unconventional secretion of FGF2 are absent from most FGF family members carrying signal peptides for ER/Golgi-dependent protein secretion

A sequence alignment of the FGF protein family covering residues 32 to 105 from FGF2 was used to identify residues with a potential role in unconventional secretion of FGF2. Residues highlighted in yellow represent cysteine residues in FGF2 that are either fully conserved throughout the FGF family (C33 and C100) or are uniquely present in FGF2 (C77 and C95). Similar to C77 and C95, K54 and K60 highlighted in green are absent from most FGF family proteins carrying signal peptides.

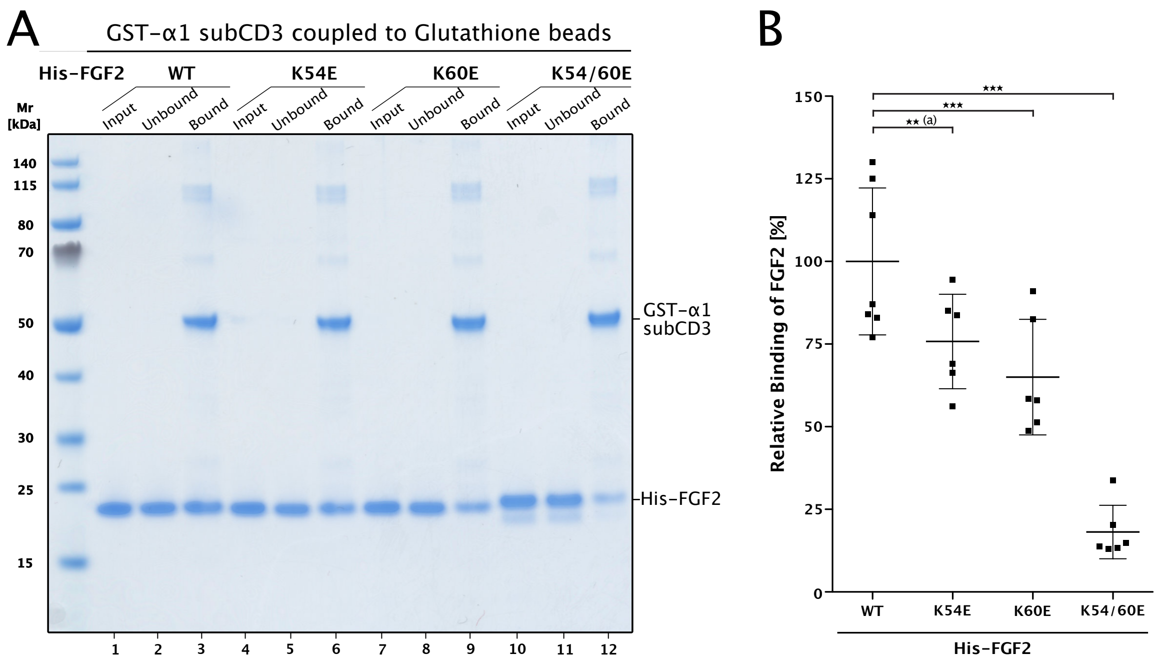

Supplementary Figure 3: K54 and K60 are required for efficient binding of FGF2 to α1-subCD3 as shown by biochemical pull-down experiments

A) Glutathione beads containing GST-tagged α1-subCD3 were incubated with His-tagged forms of FGF2 as indicated. FGF2 input (2.5%), unbound material (2.5%) and bound FGF2 (33%) were analyzed by SDS-PAGE and Coomassie Blue protein staining. Lanes 1-3: FGF2-wt; Lanes 4-6: FGF2-K54E; Lanes 7-9: FGF2-K60E; Lanes 10-12: FGF2-K54/60E. For details see Material and Methods.

B) Quantification and statistical analysis of relative binding efficiencies of FGF2-wt and mutant forms to α1-subCD3. The intensities of the FGF2 protein bands from panel A were analyzed with the ImageStudio software package (LI-COR Biosciences). Ratios of bound and unbound material were calculated and normalized to FGF2-wt. Data are shown as mean ± SD (n=6). P-value for (a) was 0.0711. *** = $p \leq 0.001$.

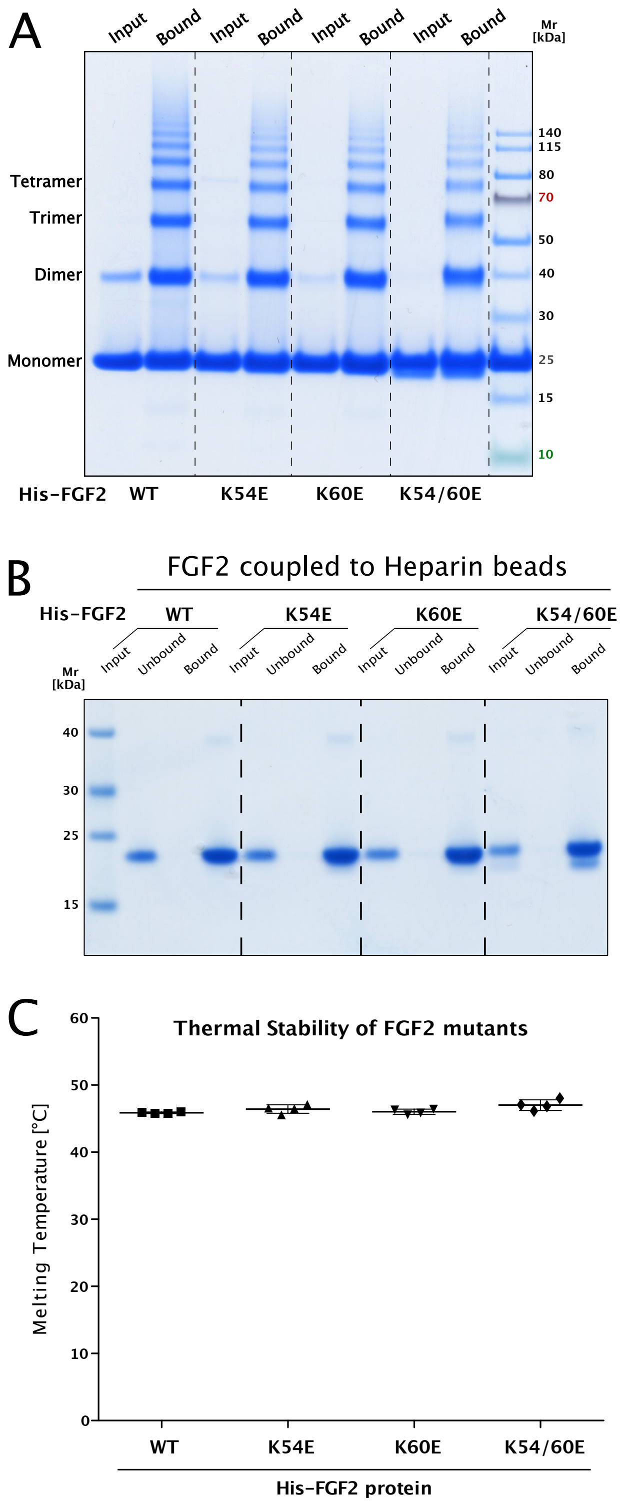

A
Input
Bound
Mr [kDa]
Tetramer
Trimer
Dimer
Monomer
140
115
80
70
50
40
30
25
15
10
His-FGF2
WT
K54E
K60E
K54/60E
B
FGF2 coupled to Heparin beads
His-FGF2
WT
K54E
K60E
K54/60E
Input
Unbound
Bound
Mr [kDa]
40
30
25
15
C
Thermal Stability of FGF2 mutants
Melting Temperature [°C]
60
50
40
30
20
10
0
WT
K54E
K60E
K54/60E
His-FGF2 protein

Supplementary Figure 4:

A) Analysis of $PI(4,5)P_2$-dependent oligomerization of the FGF2 variant forms indicated. For each FGF2 form, both input material and material bound to $PI(4,5)P_2$-containing liposomes is shown. Samples were analyzed by non-reducing SDS-PAGE and Coomassie InstantBlue protein staining. For details, see Materials and Methods.

B) Analysis of FGF2 binding to heparin for the variant forms indicated. Following incubation with heparin beads, samples were washed extensively and protein bound to beads was analyzed by reducing SDS-PAGE and Coomassie InstantBlue protein staining. Input (2.5%), unbound material (2.5%) and bound material (33%) is shown for each of the FGF2 variant forms indicated. For details, see Materials and Methods.

C) Analysis of protein folding measuring thermal stability of FGF2 variant forms. Protein samples of 10 µl at a final concentration of 3 mg/ml were analyzed by differential scanning fluorimetry (nanoDSF; [1]). Data are shown as mean ± SD (n=4). For details, see Materials and Methods.

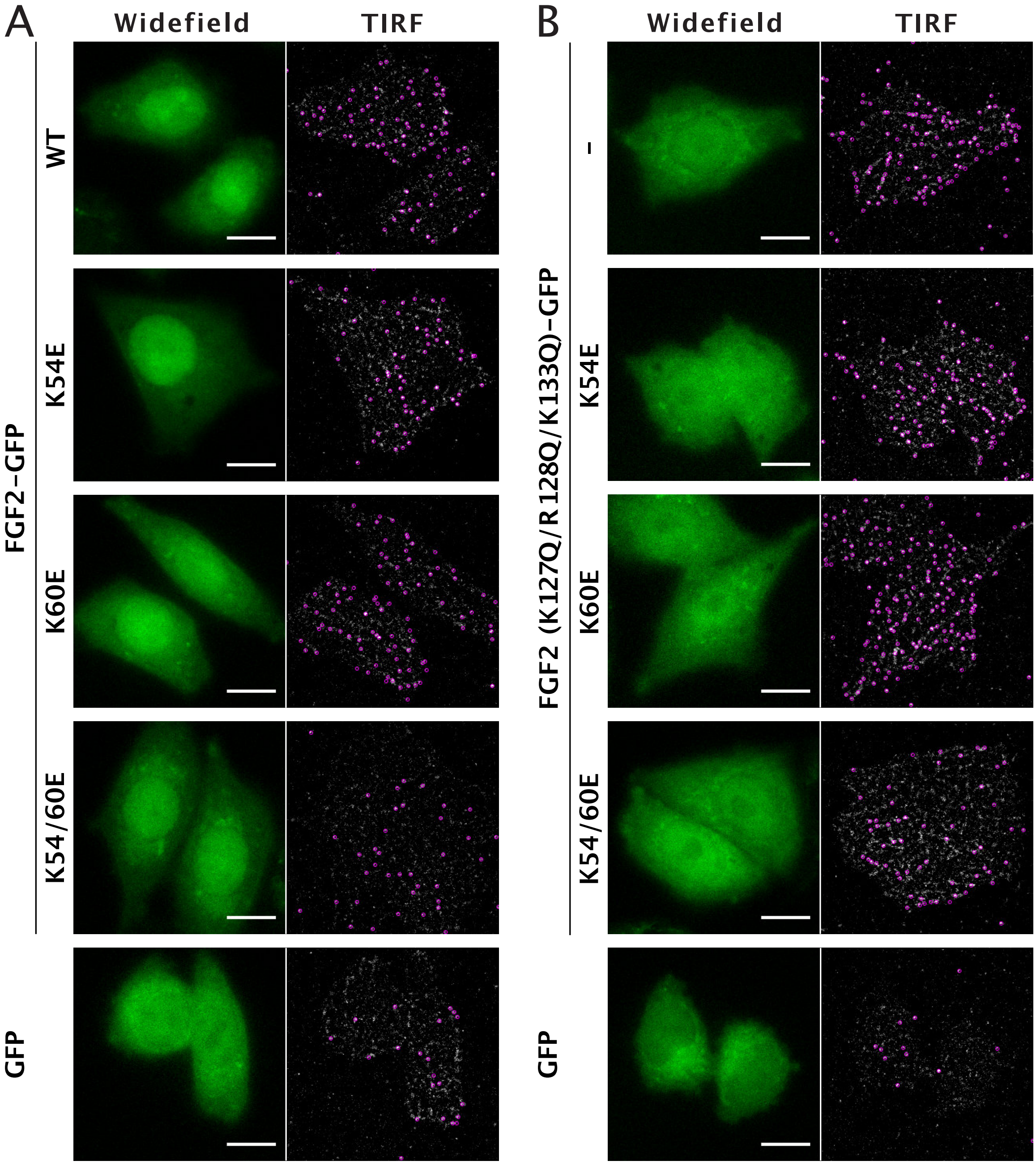
A
Widefield
TIRF
FGF2-GFP
WT
K54E
K60E
K54/60E
GFP
B
Widefield
TIRF
FGF2 (K127Q/R128Q/K133Q)-GFP
–
K54E
K60E
K54/60E
GFP

Supplementary Figure 5: Quantitative analysis of FGF2-GFP membrane recruitment in living cells at the single particle level

A) Stable CHO-K1 cell lines expressing either FGF2-wt-GFP, FGF2-K54E-GFP, FGF2-K60E-GFP, FGF2-K54/60E-GFP or GFP in a doxycycline-dependent manner were imaged employing high resolution TIRF microscopy as reported previously [2]. Single FGF2-GFP or GFP particles were identified at the inner plasma membrane leaflet (labeled by pink circles). For each cell line, a widefield image and the first frame of the corresponding TIRF video are shown. Scale bar = 10 µm.

B) Stable CHO-K1 cell lines expressing either FGF2-wt-GFP, FGF2-K127Q/R128Q/K133Q-GFP, FGF2-K54E/K127Q/R128Q/K133Q-GFP, FGF2-K60E/K127Q/R128Q/K133Q-GFP, FGF2-K54/60E/K127Q/R128Q/K133Q-GFP or GFP in a doxycycline-dependent manner were cultured and imaged as described in panel A.

Supplementary Methods

Structural analyses using NMR spectroscopy

NMR spectra were recorded at 300K on a Bruker Avance III 700MHz spectrometer equipped with a 5mm triple resonance cryo-probe. ^{1}H-^{15}N-HSQC spectra were acquired of 77 µM ^{15}N-labeled FGF2-C77/95S in the absence or presence of 70 µM or 138 µM α1-subCD3 in 25 mM HEPES (pH 7.4), 150mM KCl, 10% D_2O with 108 scans and 1024 data points in the ^{1}H and 96 data points in the ^{15}N dimension. Spectra were processed with Topspin3.2 (Bruker, Billerica, USA) and analyzed using CcpNMR Analysis 2.4.2 [3]. Peak assignments were transferred from published data [BMRB entry 4091; [4]] to nearest neighbors in recorded spectra, if possible, leading to an assignment of 63% of the non-Proline residues. Chemical shift differences were calculated using the formula $\Delta\delta(^{1}H,^{15}N)=(\delta(^{1}H)^{2}+(0.15*\delta(^{15}N))^{2})^{1/2}$. Signal-to-noise ratios were calculated from peak intensities and average noise levels. Overlapping peaks were omitted from the analysis.

Multiple sequence alignment

In order to identify residues that are uniquely present in FGF2 compared to signal-peptide-containing FGF family members, a multiple sequence alignment was performed using the EMBL-EBI MUSCLE 3.8 tool [5]. The protein sequence of isoform 3 of FGF2 (18 kDa form; UniprotKB ID P09038-2) was aligned with canonical sequences of FGF1 (P05230), FGF3 (P11487), FGF4 (P08620), FGF5 (P12034), FGF6 (P10767), FGF7 (P21781), FGF8 (P55075), FGF9 (P31371), FGF10 (O15520), FGF11 (Q92914), FGF12 (P61328), FGF13 (Q92913), FGF14 (Q92915), FGF16 (O43320), FGF17 (O60258), FGF18 (O76093), FGF19 (O95750), FGF20 (Q9NP95), FGF21 (Q9NSA1), FGF22 (Q9HCT0) and FGF23 (Q9GZV9). Supplementary Figure 2 shows the part of the alignment that covers the residues 32 to 105 of FGF2.

Biochemical pull-down experiments

For this set of experiments, Glutathione Sepharose beads were equilibrated in PBS buffer and incubated with the GST-tagged variants of α1 as indicated. GST alone was used as a negative control. GST-coupled Sepharose beads were blocked with 3% (w per v) BSA in PBS supplemented with 1 mM Benzamidine and 0.05% (w per v) Tween 20 (buffer A), washed extensively in buffer A. Finally, beads were resuspended with five bed volumes of buffer A. Per experimental condition, 75 µl of beads solution were incubated with 15 µg of His-tagged FGF2 in a total volume of 200 µl of buffer A for one hour at room temperature. Following collection of beads by low-speed centrifugation and extensive washing with buffer A, bound protein was eluted with SDS sample buffer. Both bound (33%) and unbound (2.5%) material was analyzed by SDS-PAGE followed by protein staining using Coomassie InstantBlue. Gels were scanned using the Odyssey® CLx Imaging System (LI-COR Biosciences) and band intensities were quantified using the LI-COR ImageStudio software. For quantification, the ratios between bound and unbound material were calculated for each experimental condition as indicated.

Biochemical protein-protein interaction assays

To test the ability of FGF2 variant forms to bind to $PI(4,5)P_2$ concomitant with oligomerization, FGF2 (10µM) was incubated with liposomes (2 mM total lipid) with a plasma-membrane-like lipid composition containing 3 mol% of $PI(4,5)P_2$. As described previously [6,7], experiments were conducted in a final volume of 50 µl HK-buffer (150 mM KCl, 25 mM HEPES, pH 7.4) in an Eppendorf Thermomixer at 25°C at 500rpm. After 4 hours of incubation liposomes were sedimented for 10 min at 16.000g, and washed with 100µl HK-buffer. Pellets were dissolved in 20 µl non-reducing SDS sample buffer and heated for 10 min at 65°C. Samples were analyzed on 1.0 mm NuPAGE 4-12% non-reducing Bis-Tris gels/MES-running buffer (Invitrogen) and proteins were stained with Coomassie InstantBlue (Expedeon). Input lanes contained 50% of FGF2 used in the oligomerization assays.

FGF2 variant forms were further compared to FGF2-wt with regard to their ability to bind to heparin. Heparin Sepharose 6 Fast Flow (GE Healthcare) beads (10 µl slurry) were washed four times in PBS and incubated for 1 hour at room temperature with the FGF2 variant forms indicated (15µg protein each in a total volume of 200 µl PBS). Following sedimentation of the beads and extensive washing, binding efficiency was analyzed by SDS-PAGE and protein staining using Coomassie InstantBlue (Expedeon).

To determine native protein folding by means of thermal stability, the various FGF2 and α1 variant forms used in this study were analyzed in nanoDSF experiments using a Prometheus NT 48 instrument (Nanotemper) [1]. This procedure monitors the light absorbance of proteins at 330nm and 350nm along a thermal gradient. The ratio of A_{330nm}/A_{350nm} was plotted as a function of the temperature. This allows for determining the unfolding transition midpoint, i.e. the melting temperature of a protein. The FGF2 and α1 variant forms indicated were used in a volume of 10 µl at a final concentration of 1.5 mg per ml.

Single molecule TIRF microscopy

Widefield fluorescence and TIRF images were acquired using an Olympus IX81 xCellence TIRF microscope equipped with an Olympus PLAPO 100x/1.45 Oil DIC objective lens and a Hamamatsu ImagEM Enhanced (C9100-13) camera. GFP fluorescence was excited with an Olympus 488 nm, 100 mW diode laser. Data were recorded and exported in Tagged Image File Format (TIFF) and analyzed via Fiji [8].

For the quantification of FGF2-GFP recruitment at the inner leaflet of the plasma membrane, cells were seeded in µ-Slide 8 Well Glass Bottom (ibidi) 24 h before live cell imaging experiments. The quantification of FGF2-GFP particles recruitment to the plasma membrane was achieved through the analysis of time-lapse TIRF videos. The frame of each cell was selected by widefield imaging. The number of FGF2-GFP particles were normalized to the cell surface area (μm^2) and to the expression level of FGF2-GFP. The latter was quantified at the first frame of each time-lapse TIRF video (for each analyzed cell) using ImageJ. The total number of FGF2-GFP particles per cell was quantified employing the Fiji plugin TrackMate [9]. Background fluorescence was subtracted in all the representative images and videos shown.

Supplementary References

1. Alexander, C.G. et al. Novel microscale approaches for easy, rapid determination of protein stability in academic and commercial settings. *Biochim Biophys Acta* **1844**, 2241-50 (2014).

2. Dimou, E. et al. Single event visualization of unconventional secretion of FGF2. *J Cell Biol* (2019).

3. Vranken, W.F. et al. The CCPN data model for NMR spectroscopy: development of a software pipeline. *Proteins* **59**, 687-96 (2005).

4. Moy, F.J., Seddon, A.P., Campbell, E.B., Bohlen, P. & Powers, R. 1H, 15N, 13C and 13CO assignments and secondary structure determination of basic fibroblast growth factor using 3D heteronuclear NMR spectroscopy. *J Biomol NMR* **6**, 245-54 (1995).

5. Chojnacki, S., Cowley, A., Lee, J., Foix, A. & Lopez, R. Programmatic access to bioinformatics tools from EMBL-EBI update: 2017. *Nucleic Acids Res* **45**, W550-W553 (2017).

6. Steringer, J.P. et al. Phosphatidylinositol 4,5-Bisphosphate (PI(4,5)P2)-dependent Oligomerization of Fibroblast Growth Factor 2 (FGF2) Triggers the Formation of a Lipidic Membrane Pore Implicated in Unconventional Secretion. *J Biol Chem* **287**, 27659-69 (2012).

7. Temmerman, K. et al. A direct role for phosphatidylinositol-4,5-bisphosphate in unconventional secretion of fibroblast growth factor 2. *Traffic* **9**, 1204-17 (2008).

8. Schindelin, J. et al. Fiji: an open-source platform for biological-image analysis. *Nat Methods* **9**, 676-82 (2012).

9. Tinevez, J.Y. et al. TrackMate: An open and extensible platform for single-particle tracking. *Methods* **115**, 80-90 (2017).
