# Supplementary material for: The Na,K-ATPase acts upstream of phosphoinositide PI(4,5)P2 facilitating unconventional secretion of Fibroblast Growth Factor 2
Source: Commun Biol. 2020 Mar 25;3:141. doi: 10.1038/s42003-020-0871-y (PMC7096399; doi:10.1038/s42003-020-0871-y)
Supplement: Supplementary file 7 — Reporting Summary [file 42003_2020_871_MOESM7_ESM.pdf]

nature research

Corresponding author(s): Walter Nickel

Last updated by author(s): Feb 19, 2020

Reporting Summary

Nature Research wishes to improve the reproducibility of the work that we publish. This form provides structure for consistency and transparency in reporting. For further information on Nature Research policies, see Authors & Referees and the Editorial Policy Checklist.

Statistics

For all statistical analyses, confirm that the following items are present in the figure legend, table legend, main text, or Methods section.

n/a	Confirmed	
☐	☒	The exact sample size (*n*) for each experimental group/condition, given as a discrete number and unit of measurement
☐	☒	A statement on whether measurements were taken from distinct samples or whether the same sample was measured repeatedly
☐	☒	The statistical test(s) used AND whether they are one- or two-sided<br>*Only common tests should be described solely by name; describe more complex techniques in the Methods section.*
☒	☐	A description of all covariates tested
☒	☐	A description of any assumptions or corrections, such as tests of normality and adjustment for multiple comparisons
☐	☒	A full description of the statistical parameters including central tendency (e.g. means) or other basic estimates (e.g. regression coefficient) AND variation (e.g. standard deviation) or associated estimates of uncertainty (e.g. confidence intervals)
☒	☐	For null hypothesis testing, the test statistic (e.g. *F*, *t*, *r*) with confidence intervals, effect sizes, degrees of freedom and *P* value noted<br>*Give P values as exact values whenever suitable.*
☒	☐	For Bayesian analysis, information on the choice of priors and Markov chain Monte Carlo settings
☒	☐	For hierarchical and complex designs, identification of the appropriate level for tests and full reporting of outcomes
☒	☐	Estimates of effect sizes (e.g. Cohen's *d*, Pearson's *r*), indicating how they were calculated

Our web collection on statistics for biologists contains articles on many of the points above.

Software and code

Policy information about availability of computer code

Data collection	Molecular dynamics simulation and protein docking: GROMACS 2018.3 and Rosetta 2018 packages.<br>ALPHA Assays: data from an EnVision plate reader.<br>NanoDSF : data from a Prometheus NT 48.<br>TIRF Microscope: Olympus IX81 xCellence.<br>Confocal microscope (Duolink): Nikon A1R.
Data analysis	Statistical analysis carried out on GraphPad Prism v6.<br>Intensity of bands (Pulldown and western blots) analysed on ImageStudio v5.2.5<br>Analysis of TIRF particles: Fiji with TrackMate plugin.<br>Analysis of DuoLink signal: Duolink ImageTool<br>Analysis of MD simulations and docking studies: GROMACS 2018.3.<br>Contact maps coodes available on GitHub https://gitlab.com/fabiolol/contact-probability-map/tree/master.

For manuscripts utilizing custom algorithms or software that are central to the research but not yet described in published literature, software must be made available to editors/reviewers. We strongly encourage code deposition in a community repository (e.g. GitHub). See the Nature Research guidelines for submitting code & software for further information.

Data

Policy information about availability of data

All manuscripts must include a data availability statement. This statement should provide the following information, where applicable:

- Accession codes, unique identifiers, or web links for publicly available datasets
- A list of figures that have associated raw data
- A description of any restrictions on data availability

A supplementary file with all raw data and statistical analyses contained in this work has been published along with the main publication and is available online. For

all further queries, please contact the corresponding author (Walter Nickel; walter.nickel@bzh.uni-heidelberg.de)

Field-specific reporting

Please select the one below that is the best fit for your research. If you are not sure, read the appropriate sections before making your selection.

☒ Life sciences ☐ Behavioural & social sciences ☐ Ecological, evolutionary & environmental sciences

For a reference copy of the document with all sections, see nature.com/documents/nr-reporting-summary-flat.pdf

Life sciences study design

All studies must disclose on these points even when the disclosure is negative.

Sample size	The sample size varied depending on the type of experiments and is given in the figure legends for all experiments contained in this study.
Data exclusions	In some cases, data were excluded from analysis for experiments in which technical problems occured that rendered the corresponding data inconclusive.
Replication	All experiments were conducted with biological replicates. Their number varied depending on the type of experiments and is given in the figure legends for all experiments contained in this study.
Randomization	n/a
Blinding	n/a

Reporting for specific materials, systems and methods

We require information from authors about some types of materials, experimental systems and methods used in many studies. Here, indicate whether each material, system or method listed is relevant to your study. If you are not sure if a list item applies to your research, read the appropriate section before selecting a response.

Materials & experimental systems

n/a	Involved in the study	
☐	☒	Antibodies
☐	☒	Eukaryotic cell lines
☒	☐	Palaeontology
☒	☐	Animals and other organisms
☒	☐	Human research participants
☒	☐	Clinical data

Methods

n/a	Involved in the study	
☒	☐	ChIP-seq
☒	☐	Flow cytometry
☒	☐	MRI-based neuroimaging

Antibodies

Antibodies used	mouse anti-FGF2 (clone bFM-1, Millipore)<br>rabbit anti-FGF2 (Zehe et al, 2006)<br>rabbit anti-GFP (internal)<br>mouse anti-GAPDH (Invitrogen)<br>goat secondary anti-mouse AlexaFluor680 (Invitrogen)<br>goat secondary anti-rabbit AlexaFluor800 CW (Licor)<br>rabbit anti-Na,K-ATPase alpha-1 (Zacherl et al., 2014)<br>mouse anti-Na,K-ATPase alpha-1 (Abcam 7671)
Validation	No further validation as part of this study.

Eukaryotic cell lines

Policy information about cell lines

Cell line source(s)	CHO K1 and HeLa S3 from ATCC.
Authentication	No further authentification as part of this study.
Mycoplasma contamination	All cell lines were tested and found negative for mycoplasma contamination.

Commonly misidentified lines<br>(See ICLAC register)	N/A
